# Importance of Van der Waals Interactions in Hydrogen Adsorption on a Silicon-carbide Nanotube Revisited with vdW-DFT and Quantum Monte Carlo

**DOI:** 10.1021/acsomega.1c03318

**Published:** 2021-09-16

**Authors:** Genki I. Prayogo, Hyeondeok Shin, Anouar Benali, Ryo Maezono, Kenta Hongo

**Affiliations:** † School of Information Science, JAIST, Asahidai 1-1, Nomi, Ishikawa 923-1292, Japan; ‡ Computational Science Division, Argonne National Laboratory, Argonne, Illinois 60439, United States; § Research Center for Advanced Computing Infrastructure, JAIST, Asahidai 1-1, Nomi, Ishikawa 923-1292, Japan

## Abstract

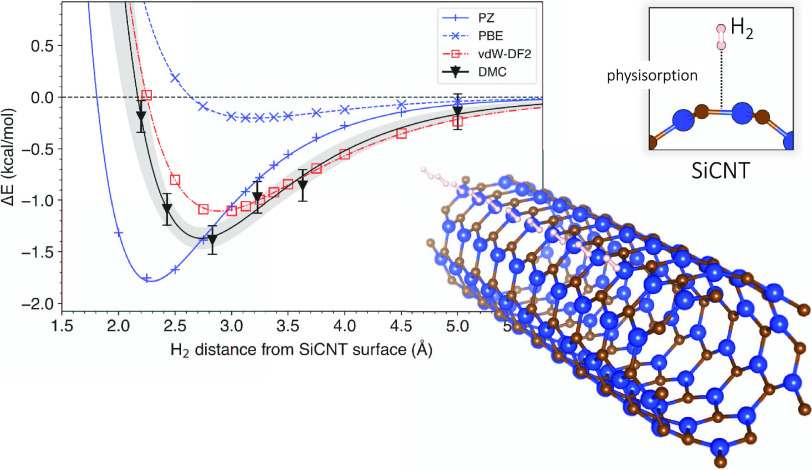

Density functional theory (DFT) is a valuable tool for calculating adsorption energies toward designing materials for hydrogen storage. However, dispersion forces being absent from the local/semi-local theory, it remains unclear as to how the consideration of van der Waals (vdW) interactions affects such calculations. For the first time, we applied diffusion Monte Carlo (DMC) to evaluate the adsorption characteristics of a hydrogen molecule on a (5,5) armchair silicon-carbide nanotube (H_2_-SiCNT). Within the DFT framework, we benchmarked various exchange-correlation functionals, including those recently developed for treating dispersion or vdW interactions. We found that the vdW-corrected DFT methods agree well with DMC, whereas the local (semilocal) functional significantly over (under)-binds. Furthermore, we fully optimized the H_2_-SiCNT geometry within the DFT framework and investigated the correlation between the structure and charge density. The vdW contribution to the adsorption was found to be non-negligible at ∼1 kcal/mol per hydrogen molecule, which amounts to 9–29% of the ideal adsorption energy required for hydrogen storage applications.

## 1 Introduction

Hydrogen energy is a promising energy resource for reducing greenhouse gas emissions.^1−3^ To realize the industrial use of hydrogen energy, particularly in the transportation sector, one of the most important developmental challenges is addressing the related storage issues—safety and capacity.^2^ Several material-based strategies to store hydrogen have been proposed, which involve choices such as the form of the stored hydrogen (physical vs. chemical storage) and the structure of the storage material (e.g., nanostructures and metal hydrides). To achieve adsorption-based room-temperature storage, the ideal interaction energy between the stored hydrogen and storage material has been estimated as ∼3.5–11.5 kcal/mol.^4,5^ At the moment, this technology is limited to low-temperature storage because the interaction is too weak to resist, being overpowered by thermal energy at the desired higher temperatures. Computational material design would be immensely helpful in further exploring appropriate storage materials from the massive material space, provided hydrogen adsorption energies on candidate materials can be accurately predicted at reasonable computational costs.

Silicon-carbide nanotubes (SiCNTs) are a typical nanostructure studied for the above purpose owing to their enhanced molecular interactions compared with those of the structurally related (and more common) carbon nanotubes (CNTs). This feature has been linked to the SiCNT polarized surface originating from the Si–C bonds.^6−9^ Despite this, density functional studies based on local (LDA), semilocal (GGA), and hybrid (B3LYP) exchange-correlation (XC) functionals on pristine nanotubes estimated the adsorption energy of hydrogen between 0.7 and 1.98 kcal/mol,^10−13^ which is still too small for storing hydrogen at the desired ambient temperature. This has motivated further investigations into doping schemes with dopants such as alkali and transition metals^12,14^ or with vacancies.^11,15^ These studies, however, did not incorporate van der Waals (vdW) corrections into the conventional DFT scheme, and thus they may have underestimated the true adsorption potential. From another viewpoint, the basis sets adopted for DFT calculations also matter, since Gaussian and numerical basis sets can give rise to overestimated interaction energies owing to the basis set superposition error.^16^ Thus, the true adsorption potential of SiCNTs remains unclear, even when the results seem plausible and are consistent with the experimentally observed higher hydrogen uptake than that of CNTs.^17,18^

In this study, we demonstrate the importance of incorporating vdW interactions for a quantitative description of H_2_ adsorption on SiCNTs and related systems. We benchmarked leading vdW-corrected functionals for DFT against diffusion Monte Carlo (DMC) and conventional XCs in terms of binding curves and charge densities. DMC^19^ is a stochastic method in which an “exact” many-body Hamiltonian (without any approximation) is used to project a set of electron positions into their ground-state configuration, allowing dispersion forces and dynamical correlations to be rigorously captured without the use of additional corrections. In this approach, DFT is only used to construct the initial Slater determinant from its Kohn–Sham orbitals and otherwise has only minimal effect on the final results.^20^ DMC has been proved to be highly accurate for various noncovalent systems^20−31^ and is comparable to the best correlated methods, while offering a better computational cost and minimal systematic error.^20^ Finally, we investigated the fully optimized H_2_-SiCNT geometries for our benchmark set of XC functionals and discussed their relationship to the charge densities at a fixed geometry, along with the implications of their difference in terms of the charge densities.

## 2 Computational Details

### 2.1 Structural Modeling

**Figure 1:**
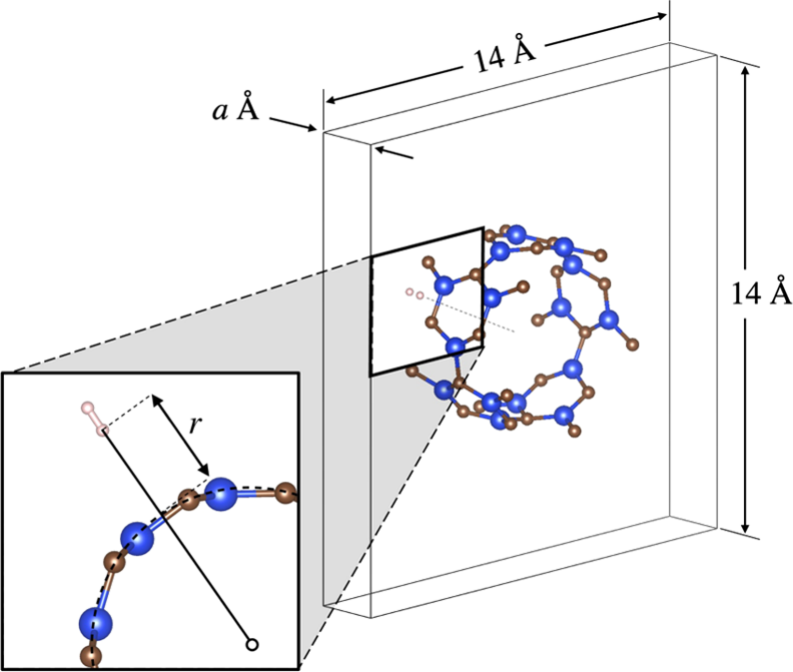
Vertically oriented hydrogen molecule over an SiCNT hollow site. Inset: the distance is measured over the largest diameter of the SiCNT, which corresponds to the C site.

We selected the type-I (5,5) armchair SiCNT with 20 atoms per primitive cell. Consisting only of Si–C bonds, type-I was found to be more stable than those containing Si–Si and C–C bonds.^7,32,33^ The basic structure was built using a generic nanotube generation tool^34^ and later optimized in DFT. A 14 × 14 × *a* Å simulation cell was used to minimize spurious interactions with periodic images, and the periodic unit length (*a*) was optimized along with the other geometries. For benchmark, we considered only the vertical mode of a single hydrogen molecule over the hollow site of the SiCNT (Figure 1). We discuss the effect of performing full optimization of hydrogen orientation in the later part of the results. The distance was varied to obtain a binding curve for which the energies were least-square-fitted to a Morse potential. The binding energy corresponding to the energy change from the free to the bound configuration is defined as
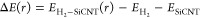
1Each component on the right-hand side was computed independently using the same simulation cell. Since the diameter of the SiCNT is not singular due to lengthwise buckling, we define *r* as the distance from the hydrogen molecule to the center of the nanotube subtracted by the largest diameter within the primitive cell.

### 2.2 Density Functional Theory (DFT)

The PWSCF binary in the QUANTUM ESPRESSO^35^ package was used for all DFT calculations based on plane wave basis sets with pseudopotentials. The kinetic energy cutoff and *k*-point grid size were converged at 150 hartree and a 1 × 1 × 6 Monkhorst–Pack grid,^36^ respectively, to achieve chemical accuracy. To reduce the timestep error in the later DMC stage, the nonsingular energy-consistent pseudopotential reported by Burkatzki et al.^37^ was utilized for all calculations. All geometry optimizations were performed within PBE with total force and energy thresholds of 10^–10^ and 10^–4^ a.u., respectively.

We compared the vdW approaches DFT-D2, DFT-D3, Tkatchenko–Scheffler (TS), Exchange-Dipole Model (XDM), vdW-DF2, and rVV10;^38−45^ the first four are based on an explicit *R*^–6^ pairwise potential operating on atomic coordinates, whereas the latter two have an exchange-correlation potential modified by the addition of a nonlocal correlation term. The additional term in the exchange-correlation potential allows for changes in the charge density, providing more information for analyzing the binding formation. To make a comparison with previous studies,^10−13^ we contrast their performance relative to the LDA from Perdew–Zunger (PZ)^46^ and GGA from Perdew–Burke–Ernzerhof (PBE).^47^

### 2.3 Diffusion Monte Carlo (DMC)

QMCPACK^48^ was used for all quantum Monte Carlo (QMC) calculations, the main DMC calculations, and for the variational Monte Carlo (VMC) calculations used in preparation of the trial wavefunction. The trial wavefunction was a Slater–Jastrow type, comprising a single determinant with PBE-DFT orbitals and a Jastrow factor with optimized parameters at the VMC level. Parameters consisting of one- and two-body interaction terms were used, each in the form of B-splines with 10 optimizable parameters for each atom type. For efficient statistical accumulation, Jastrow parameters were optimized by minimizing VMC energy using a hybrid method mixing the linear method algorithm^49^ and an accelerated descent method as described by Otis and Neuscamann.^50^ The orbitals were pre-projected into B-splines^51^ to increase the computational efficiency. The pseudopotential parts were evaluated with the *T*-move scheme.^52^ The timestep and finite size errors were eliminated through linear extrapolation, first by the timestep and then by the real space supercell size. The DMC timesteps were 0.0025, 0.01, and 0.04 a.u, and the supercells were 2, 6, and 8 times duplicates of the primitive unit cell in the cylinder length, each with twist-averaged boundary conditions^53^ on regular grids of 1 × 1 × 8, 1 × 1 × 3, and 1 × 1 × 2, respectively. The mixed boundary conditions were applied with open boundaries on the 14 Å sides and the target walker population was set to 4096. In the Supporting Information, these potential biases in DMC are discussed and proved to be small enough for the purpose of this study.

## 3 Results and Discussion

### 3.1 Structural Properties

We found that the geometry of the (5,5) SiCNT is relatively unaffected by the choice of the XC functional (Table 1). This result is unsurprising due to its construction of only single covalent bonds involving s and p electrons. The obtained bond lengths were also consistent with those reported in prior hybrid works,^32,54^ albeit slightly shorter than those obtained utilizing cluster models.^12,33^ It is not unusual to have a slight distortion near cluster terminations that does not exist in periodic models such as the one used in this work. Thus, we conclude that the conventional XCs are sufficient to describe the SiCNT model used in this work.

**Table 1:** PZ- and PBE-Optimized Structural Parameters of a (5,5) SiCNT Given in Å^a^

XC	*R*_SiCNT_	buckling	⟨Si–C⟩	⟨Si–C_p_⟩	⟨Si–C_d_⟩
PZ	4.341	0.103	1.791	1.790	1.793
PBE	4.338	0.101	1.790	1.788	1.792

a The subscripts “p” and “d” denote perpendicular and diagonal bonds relative to the cylinder axis, respectively.

### 3.2 H_2_ Adsorption on SiCNT

**Figure 2:**
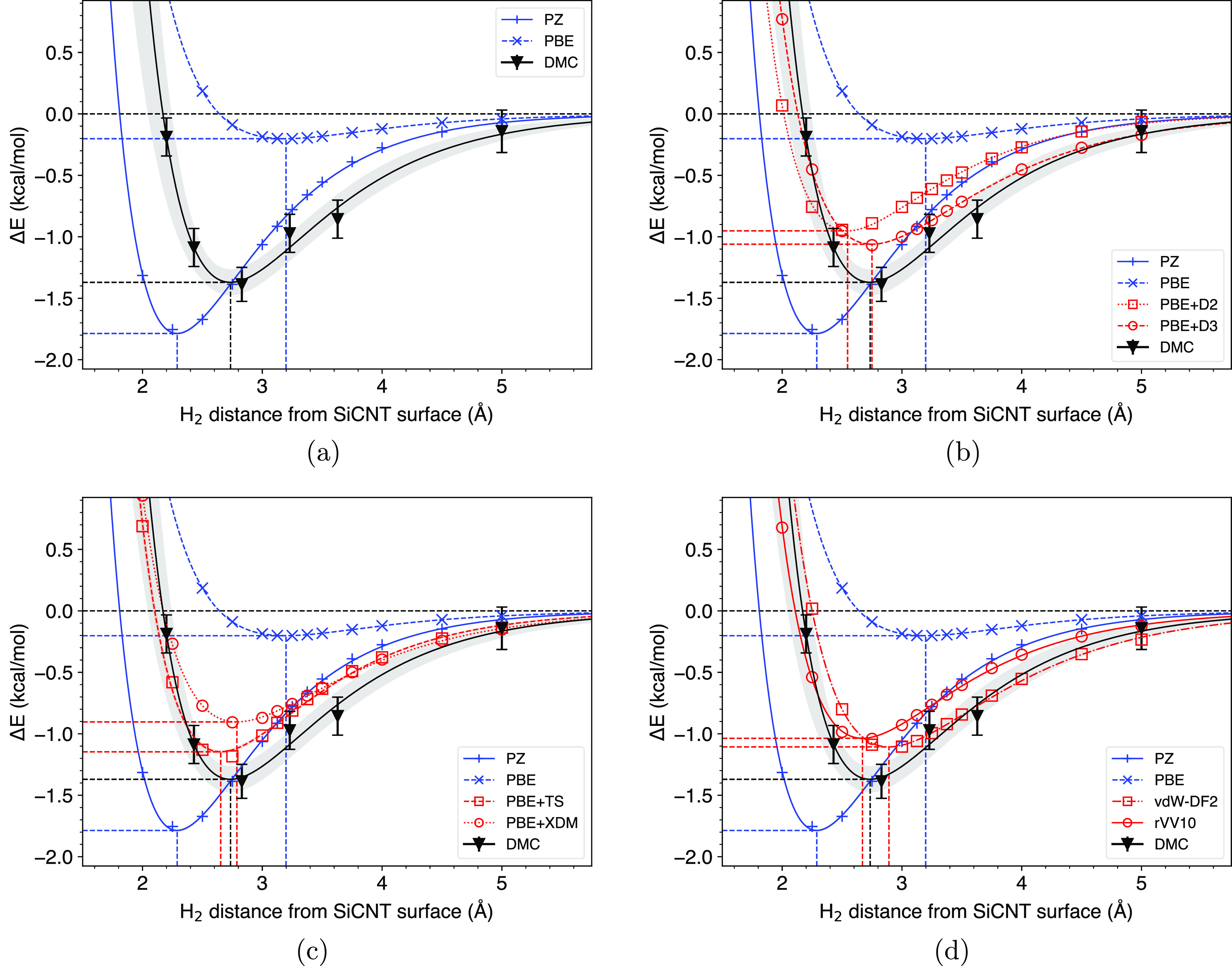
Binding energy vs H_2_ distance plot for (a) conventional XCs and DMC, compared to (b) PBE + D2 and PBE + D3, (c) PBE + TS and PBE + XDM, and (d) vdW-DF2 and rVV10. Blue curves represent conventional XCs, while red and black curves represent vdW-corrected XCs and DMC, respectively. Dashed lines indicate each fitting curve’s minimum. While energetically similar, the minima from the vdW-corrected XCs are at different positions, and they slightly underbind relative to DMC.

**Figure 3:**
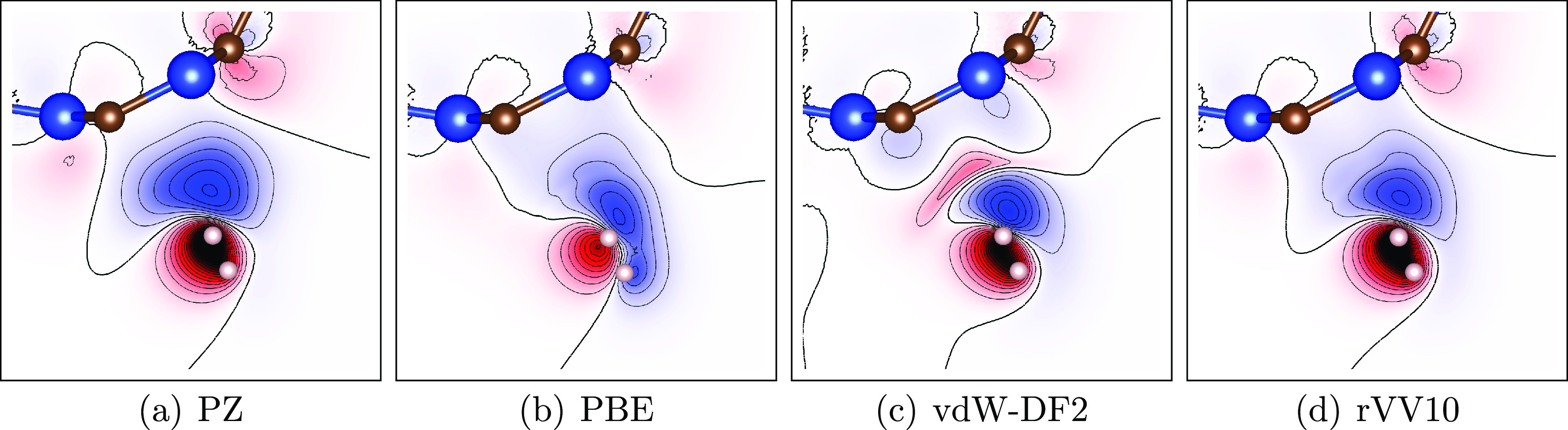
Charge density difference Δρ(*r*) in the presence of other fragments vs isolated fragments: (a) PZ, (b) PBE, (c) vdW-DF2, and (d) rVV10. See the text for the definition of Δρ(*r*). Blue denotes a positive difference, while red denotes a negative difference. The distributions from vdW-DF2 and rVV10 are notably distinct; rVV10 is similar to PZ, while vdW-DF2 shows a slight dip in between despite their similar binding curves.

The main interest is how well the adsorption curves from the vdW corrections agree with the DMC results and among themselves. As shown in Figure 2, it is clear that PZ (PBE) severely over (under)-binds the DMC target value by 0.6 (1.2) kcal/mol. This result is consistent with the established behavior of LDA and GGA in vdW-dominated systems. The nonempirical vdW-DF2 and rVV10 functionals return virtually identical binding energies but their H_2_ separations differ by ∼0.2 Å. Interestingly, the D2 energetics agree more with the nonempirical methods relative to D3. This was accompanied by a slight underestimation of the H_2_ separation distance, which was corrected by D3. Considering they both start from PBE energies, this is satisfactory. In general, all vdW corrections underbind relative to DMC but with more reasonable adsorption minima. This suggests that while the geometries derived from these functionals can generally be trusted, a more careful consideration of their energetics is necessary. Overall, PBE + TS produces the closest binding energy to the DMC, while the H_2_ distance is best reproduced by PBE + D3. LDA is known to produce spurious covalent bonding between noncovalent molecules due to the self-interaction error.^22,26^ This gives rise to ca. 0.6 kcal/mol overbinding. GGA improves the self-interaction error, but at the cost of weak intermolecular interactions as the vdW interaction is not inherently accounted for. To illustrate this point, we plotted the charge density difference between the whole and isolated components, Δρ(*r*) = ρ_SiCNT+H_2__(*r*) – ρ_SiCNT_(*r*) – ρ_H_2__(*r*), in Figure 3. The charge accumulation between the H_2_ molecule and SiCNT, i.e., spurious covalent bonding, is prominent for LDA-PZ as shown in Figure 3(a). This feature is not present in the GGA-PBE data, as shown in Figure 3(b), and is instead replaced by a much weaker redistribution of charge toward the SiCNT surface. Note that while the pairwise corrections (D2, D3, TS, and XDM) properly address the binding curve, they do not affect the charge density at a given geometry. In contrast, vdW-DF2 and rVV10 achieve their corrections by deforming the charge density (as a side effect of changes at the wavefunction level) using nonlocal perturbations in the correlation integral. As shown in Figure 3c,d for vdW-DF2 and rVV10, respectively, there is a slight dip in charge density in the shape of a bow; this is associated with the existence of noncovalent-type interactions,^55^ although it is much weaker in the rVV10 case. Interestingly, the rVV10 distribution is much closer to the PZ distribution as opposed to the distinct distribution of vdW-DF2. The lack of proper vdW interactions in PZ and PBE results in misestimation by more than 0.5 kcal/mol, with further potential for errors arising from incorrect geometry predictions. Since the target adsorption energy is 3.5–11.5 kcal/mol, this misestimation is not negligible. We thus claim that the inclusion of vdW corrections in the XC functional is essential for the quantitative evaluation of adsorption energies, particularly in the vdW-heavy H_2_-SiCNT system, and more generally for further computational exploration of storage materials (Table 2).

**Table 2:** Morse Fit for the Binding Energy vs Vertical-Oriented H_2_ Distance Plot for All XCs and DMC

XC	*R*_H_2__ [Å]	Δ*E* [kcal/mol]
PZ	2.289	1.787
PBE	3.198	0.202
PBE + D2	2.546	0.952
PBE + D3	2.750	1.060
PBE + TS	2.653	1.147
PBE + XDM	2.788	0.904
vdW-DF2	2.892	1.107
rVV10	2.670	1.037
DMC	2.735	1.370(106)

### 3.3 Fully Optimized Geometries

In Section 3.2, we varied only the distance between H_2_ and SiCNT for our benchmark purpose of evaluating the vdW interactions. Here, we consider the fully optimized geometries to evaluate a more realistic adsorption energy. Table 3 gives the structural and energetics information obtained from all of the DFT methods adopted in the present study. Similar to the findings discussed in Section 3.2, the vdW-corrected functionals (vdW-DF2 and rVV10) clearly give highly similar trends, while LDA (GGA) over (under)-binds, which is well-known for noncovalent systems.^26^ Within the framework of the vdW-corrected functionals, they agree well with each other in terms of the structural parameters and adsorption energy. Compared to the vdW-corrected functionals, PBE gives poor description of all of the structural and energetics properties. In contrast, LDA-PZ gives a surface angle θ_s_ closer to the nonlocal vdW corrections (vdW-DF2 and rVV10), but looking at the other properties, they are significantly different from each other; therefore, we may regard this coincidence in PZ as being accidental. These structural properties are associated with the charge densities as shown in Figure 3. Overall, LDA and the two vdW-DFT functionals (vdW-DF2 and rVV10) give different shapes than PBE. As the charge density at the bonding region between H_2_ and SiCNT increases (LDA > rVV10 > vdW-DF2), *R*_H_2__ and θ_s_ both decrease (LDA < rVV10 < vdW-DF2). As mentioned in Section 3.2, the LDA bonding is not noncovalent but spurious. It is evident from the correlation between the charge density and structure that the LDA overbinding correlates to the highest charge density at the bonding region.

**Table 3:** Optimized H_2_ Conformations and Adsorption Energies from All Tested XC Functionals^a^

XC	*R*_H_2__ [Å]	θ_s_ [deg.]	*E*_ads_ [kcal/mol]
PZ	2.573	51.3	2.375
PBE	3.437	37.6	0.291
PBE + D2	2.792	45.3	1.333
PBE + D3	3.000	45.7	1.421
PBE + TS	2.872	52.4	1.592
PBE + XDM	3.037	50.7	1.152
vdW-DF2	3.170	54.4	1.268
rVV10	2.930	53.9	1.298

a The energy values are from full optimization calculations.

Herein, we compare the pairwise vdW corrections to the vdW-corrected functionals. In particular, the surface angle θ_*s*_ values obtained from the “empirical” pairwise ones (PBE-D2/D3) improve the PBE value, but they are still smaller than those from the vdW-corrected functionals. This can be attributed to the fact that the empirical corrections never deform the charge densities (or equivalently wave functions) self-consistently as the total energies just decrease due to the empirical pair potentials. In other words, the charge densities of the pairwise vdW corrections (PBE-D2/D3) are the same as those of their underlying XC functional (PBE). In contrast, since the other pairwise vdW corrections (PBE-TS and -XDM) can take the local chemical environment into account, they significantly improve the θ_*s*_ values compared to the PBE value, though they still underestimate slightly compared to the vdW-corrected functionals. As for the other properties, PBE + TS overestimates the adsorption energy (*E*_ads_) and underestimates the bond length (*R*_H_2__), while PBE + XDM give *E*_ads_ and *R*_H_2__ closer to the vdW-corrected functionals (**R18**). Considering the above findings, we may conclude that the self-consistent charge deformation is important, and pairwise vdW correction alone is insufficient for an accurate structure determination.

## 4 Conclusions

We performed DMC and various DFT simulations to evaluate the adsorption energies of a hydrogen molecule on a SiCNT with a (5,5) armchair structure. Recently developed XC functionals designed to reproduce vdW interactions (PBE + D3, vdW-DF2, and rVV10) and conventional XC functionals (PZ, PBE) were compared to DMC as a reference. Overall, all of the vdW-corrected XC functionals agree well with DMC, whereas PZ (PBE) over (under)-binds. The self-consistent nonlocal correlation functionals, vdW-DF2 and rVV10, give almost the same adsorption energies. Differences in the structural properties were found to closely correlate with differences in the charge density distribution. A higher charge density in the bonding region leads to a shorter distance between H_2_ and SiCNT and larger surface angle. The magnitude of the vdW interaction was estimated to be ca. 1.2 kcal/mol, which corresponds to 9–29% of the ideal adsorption energy for hydrogen storage. This finding implies the importance of vdW corrections within the framework of DFT. We thus conclude that protocols based on vdW-corrected XC functionals will advance the computational investigation and exploration of storage materials in the near future. In addition, our results support previous findings that pristine SiCNT alone is unlikely to provide a sufficient binding for realizing ambient temperature hydrogen adsorption; thus, further research studies into doping and other surface modifications should be pursued to use it as the basis for a hydrogen storage system.

## Supporting Information Available

DMC energies, finite size error corrections, and timestep extrapolation plots (PDF)

## References

[ref1] Dincer I. Technical, environmental and exergetic aspects of hydrogen energy systems. Int. J. Hydrogen Energy 2002, 27, 265–285. 10.1016/S0360-3199(01)00119-7.

[ref2] Eberle U.; Felderhoff M.; Schüth F. Chemical and Physical Solutions for Hydrogen Storage. Angew. Chem., Int. Ed. 2009, 48, 6608–6630. 10.1002/anie.200806293. 19598190

[ref3] Gröger O.; Gasteiger H. A.; Suchsland J.-P. Review—Electromobility: Batteries or Fuel Cells. J. Electrochem. Soc. 2015, 162, A2605–A2622. 10.1149/2.0211514jes.

[ref4] Jhi S.-H. Activated boron nitride nanotubes: A potential material for room-temperature hydrogen storage. Phys. Rev. B 2006, 74, 155424 10.1103/PhysRevB.74.155424.

[ref5] Bhatia S. K.; Myers A. L. Optimum Conditions for Adsorptive Storage. Langmuir 2006, 22, 1688–1700. 10.1021/la0523816. 16460092

[ref6] Mavrandonakis A.; Froudakis G. E.; Schnell M.; Mühlhäuser M. From Pure Carbon to Silicon-Carbon Nanotubes: An Ab-initio Study. Nano Lett. 2003, 3, 1481–1484. 10.1021/nl0343250.

[ref7] Alam K. M.; Ray A. K. A hybrid density functional study of zigzag SiC nanotubes. Nanotechnology 2007, 18, 495706 10.1088/0957-4484/18/49/495706. 20442487

[ref8] Baumeier B.; Krüger P.; Pollmann J. Structural, elastic, and electronic properties of SiC, BN, and BeO nanotubes. Phys. Rev. B 2007, 76, 085407 10.1103/PhysRevB.76.085407.

[ref9] Barghi S. H.; Tsotsis T. T.; Sahimi M. Chemisorption, physisorption and hysteresis during hydrogen storage in carbon nanotubes. Int. J. Hydrogen Energy 2014, 39, 1390–1397. 10.1016/j.ijhydene.2013.10.163.

[ref10] Mpourmpakis G.; Froudakis G. E.; Lithoxoos G. P.; Samios J. SiC Nanotubes: A Novel Material for Hydrogen Storage. Nano Lett. 2006, 6, 1581–1583. 10.1021/nl0603911. 16895338

[ref11] Devi N. R.; Gayathri V. Effect of structural defects on the hydrogen adsorption in promising nanostructures. Comput. Mater. Sci. 2015, 96, 284–289. 10.1016/j.commatsci.2014.09.017.

[ref12] Tabtimsai C.; Ruangpornvisuti V.; Tontapha S.; Wanno B. A DFT investigation on group 8B transition metal-doped silicon carbide nanotubes for hydrogen storage application. Appl. Surf. Sci. 2018, 439, 494–505. 10.1016/j.apsusc.2017.12.255.

[ref13] Wang X.; Liew K. M. Hydrogen Storage in Silicon Carbide Nanotubes by Lithium Doping. J. Phys. Chem. C 2011, 115, 3491–3496. 10.1021/jp106509g.

[ref14] Banerjee S.; Nigam S.; Pillai C.; Majumder C. Hydrogen storage on Ti decorated SiC nanostructures: A first principles study. Int. J. Hydrogen Energy 2012, 37, 3733–3740. 10.1016/j.ijhydene.2011.05.078.

[ref15] Baierle R. J.; Miwa R. H. Hydrogen interaction with native defects in SiC nanotubes. Phys. Rev. B 2007, 76, 205410 10.1103/PhysRevB.76.205410.

[ref16] Sheng X. W.; Mentel L.; Gritsenko O. V.; Baerends E. J. Counterpoise correction is not useful for short and van der Waals distances but may be useful at long range. J. Comput. Chem. 2011, 32, 2896–2901. 10.1002/jcc.21872. 21735451

[ref17] Barghi S. H.; Tsotsis T. T.; Sahimi M. Hydrogen sorption hysteresis and superior storage capacity of silicon-carbide nanotubes over their carbon counterparts. Int. J. Hydrogen Energy 2014, 39, 21107–21115. 10.1016/j.ijhydene.2014.10.087.

[ref18] Barghi S. H.; Tsotsis T. T.; Sahimi M. Experimental investigation of hydrogen adsorption in doped silicon-carbide nanotubes. Int. J. Hydrogen Energy 2016, 41, 369–374. 10.1016/j.ijhydene.2015.10.091.

[ref19] Foulkes W. M. C.; Mitas L.; Needs R. J.; Rajagopal G. Quantum Monte Carlo simulations of solids. Rev. Mod. Phys. 2001, 73, 33–83. 10.1103/RevModPhys.73.33.

[ref20] Dubecký M.; Mitas L.; Jurečka P. Noncovalent Interactions by Quantum Monte Carlo. Chem. Rev. 2016, 116, 5188–5215. 10.1021/acs.chemrev.5b00577. 27081724

[ref21] Korth M.; Luchow A.; Grimme S. Toward the Exact Solution of the Electronic Schrodinger Equation for Noncovalent Molecular Interactions: Worldwide Distributed Quantum Monte Carlo Calculations. J. Phys. Chem. A 2008, 112, 2104–2109. 10.1021/jp077592t. 18201073

[ref22] Hongo K.; Watson M. A.; Sánchez-Carrera R. S.; Iitaka T.; Aspuru-Guzik A. Failure of Conventional Density Functionals for the Prediction of Molecular Crystal Polymorphism: A Quantum Monte Carlo Study. J. Phys. Chem. Lett. 2010, 1, 1789–1794. 10.1021/jz100418p.

[ref23] Watson M. A.; Hongo K.; Iitaka T.; Aspuru-Guzik A. Advances in Quantum Monte Carlo; Chapter 9, pp 101–117.

[ref24] Dubecký M.; Jurečka P.; Derian R.; Hobza P.; Otyepka M.; Mitas L. Quantum Monte Carlo Methods Describe Noncovalent Interactions with Subchemical Accuracy. J. Chem. Theory Comput. 2013, 9, 4287–4292. 10.1021/ct4006739. 26589147

[ref25] Dubecký M.; Derian R.; Jurečka P.; Mitas L.; Hobza P.; Otyepka M. Quantum Monte Carlo for noncovalent interactions: an efficient protocol attaining benchmark accuracy. Phys. Chem. Chem. Phys. 2014, 16, 20915–20923. 10.1039/C4CP02093F. 25170978

[ref26] Hongo K.; Cuong N. T.; Maezono R. The importance of electron correlation on stacking interaction of adenine-thymine base-pair step in B-DNA: A quantum Monte Carlo study. J. Chem. Theory Comput. 2013, 9, 1081–1086. 10.1021/ct301065f. 26588751

[ref27] Hongo K.; Watson M. A.; Iitaka T.; Aspuru-Guzik A.; Maezono R. Diffusion Monte Carlo Study of Para-Diiodobenzene Polymorphism Revisited. J. Chem. Theory Comput. 2015, 11, 907–917. 10.1021/ct500401p. 26579744

[ref28] Hongo K.; Maezono R. Recent Progress in Quantum Monte Carlo; Chapter 9, pp 127–143.

[ref29] Hongo K.; Maezono R. A Computational Scheme To Evaluate Hamaker Constants of Molecules with Practical Size and Anisotropy. J. Chem. Theory Comput. 2017, 13, 5217–5230. 10.1021/acs.jctc.6b01159. 28981266

[ref30] Shin H.; Kim J.; Lee H.; Heinonen O.; Benali A.; Kwon Y. Nature of Interlayer Binding and Stacking of sp–sp ^2^ Hybridized Carbon Layers: A Quantum Monte Carlo Study. J. Chem. Theory Comput. 2017, 13, 5639–5646. 10.1021/acs.jctc.7b00747. 28945968

[ref31] Oqmhula K.; Hongo K.; Maezono R.; Ichibha T. Ab Initio Evaluation of Complexation Energies for Cyclodextrin-Drug Inclusion Complexes. ACS Omega 2020, 5, 19371–19376. 10.1021/acsomega.0c01059. 32803030 PMC7424588

[ref32] Alam K. M.; Ray A. K. Hybrid density functional study of armchair SiC nanotubes. Phys. Rev. B 2008, 77, 035436 10.1103/PhysRevB.77.035436.

[ref33] Menon M.; Richter E.; Mavrandonakis A.; Froudakis G.; Andriotis A. N. Structure and stability of SiC nanotubes. Phys. Rev. B 2004, 69, 115322 10.1103/PhysRevB.69.115322.

[ref34] Frey J. T.; Doren D. TubeGen 3.4. http://turin.nss.udel.edu/research/tubegenonline.html, 2011 (Accessed on 2019-01-21).

[ref35] Giannozzi P.; et al. QUANTUM ESPRESSO: a modular and open-source software project for quantum simulations of materials. J. Phys.: Condens. Matter 2009, 21, 395502 10.1088/0953-8984/21/39/395502. 21832390

[ref36] Monkhorst H. J.; Pack J. D. Special points for Brillouin-zone integrations. Phys. Rev. B 1976, 13, 5188–5192. 10.1103/PhysRevB.13.5188.

[ref37] Burkatzki M.; Filippi C.; Dolg M. Energy-consistent pseudopotentials for quantum Monte Carlo calculations. J. Chem. Phys. 2007, 126, 234105 10.1063/1.2741534. 17600402

[ref38] Dion M.; Rydberg H.; Schröder E.; Langreth D. C.; Lundqvist B. I. Van Der Waals Density Functional for General Geometries. Phys. Rev. Lett. 2004, 92, 246401 10.1103/PhysRevLett.92.246401. 15245113

[ref39] Grimme S. Semiempirical GGA-type density functional constructed with a long-range dispersion correction. J. Comput. Chem. 2006, 27, 1787–1799. 10.1002/jcc.20495. 16955487

[ref40] Grimme S.; Antony J.; Ehrlich S.; Krieg H. A consistent and accurate ab initio parametrization of density functional dispersion correction (DFT-D) for the 94 elements H-Pu. J. Chem. Phys. 2010, 132, 154104 10.1063/1.3382344. 20423165

[ref41] Lee K.; Murray E. D.; Kong L.; Lundqvist B. I.; Langreth D. C. Higher-accuracy van der Waals density functional. Phys. Rev. B 2010, 82, 081101 10.1103/PhysRevB.82.081101.

[ref42] Tkatchenko A.; Scheffler M. Accurate Molecular van der Waals Interactions from Ground-State Electron Density and Free-Atom Reference Data. Phys. Rev. Lett. 2009, 102, 073005 10.1103/PhysRevLett.102.073005. 19257665

[ref43] Otero-de-la Roza A.; Johnson E. R. Van Der Waals interactions in solids using the exchange-hole dipole moment model. J. Chem. Phys. 2012, 136, 174109 10.1063/1.4705760. 22583212

[ref44] Sabatini R.; Gorni T.; de Gironcoli S. Nonlocal van der Waals density functional made simple and efficient. Phys. Rev. B 2013, 87, 041108 10.1103/PhysRevB.87.041108.

[ref45] Thonhauser T.; Zuluaga S.; Arter C. A.; Berland K.; Schröder E.; Hyldgaard P. Spin Signature of Nonlocal Correlation Binding in Metal-Organic Frameworks. Phys. Rev. Lett. 2015, 115, 136402 10.1103/PhysRevLett.115.136402. 26451571

[ref46] Perdew J. P.; Zunger A. Self-interaction correction to density-functional approximations for many-electron systems. Phys. Rev. B 1981, 23, 5048–5079. 10.1103/PhysRevB.23.5048.

[ref47] Perdew J. P.; Burke K.; Ernzerhof M. Generalized Gradient Approximation Made Simple. Phys. Rev. Lett. 1996, 77, 3865–3868. 10.1103/PhysRevLett.77.3865. 10062328

[ref48] Kim J.; et al. QMCPACK: an open source ab initio quantum Monte Carlo package for the electronic structure of atoms, molecules and solids. J. Phys.: Condens. Matter 2018, 30, 195901 10.1088/1361-648X/aab9c3. 29582782

[ref49] Toulouse J.; Umrigar C. J. Optimization of quantum Monte Carlo wave functions by energy minimization. J. Chem. Phys. 2007, 126, 084102 10.1063/1.2437215. 17343435

[ref50] Otis L.; Neuscamman E. Complementary first and second derivative methods for ansatz optimization in variational Monte Carlo. Phys. Chem. Chem. Phys. 2019, 21, 14491–14510. 10.1039/C9CP02269D. 31245799

[ref51] Alfé D.; Gillan M. J. Efficient localized basis set for quantum Monte Carlo calculations on condensed matter. Phys. Rev. B 2004, 70, 161101 10.1103/PhysRevB.70.161101.

[ref52] Casula M.; Moroni S.; Sorella S.; Filippi C. Size-consistent variational approaches to nonlocal pseudopotentials: Standard and lattice regularized diffusion Monte Carlo methods revisited. J. Chem. Phys. 2010, 132, 154113 10.1063/1.3380831. 20423174

[ref53] Lin C.; Zong F. H.; Ceperley D. M. Twist-averaged boundary conditions in continuum quantum Monte Carlo algorithms. Phys. Rev. E 2001, 64, 016702 10.1103/PhysRevE.64.016702. 11461437

[ref54] Wang Y.; Zhao C.; Su K.; Wang X.; Qin X.; Yuan Z. A Density Functional Theoretical Study on Ultra Long Armchair (n,n ) Single Walled Carbon Silicon Nanotubes. J. Nanosci. Nanotechnol. 2017, 17, 3809–3815. 10.1166/jnn.2017.13995.

[ref55] Contreras-García J.; Johnson E. R.; Keinan S.; Chaudret R.; Piquemal J.-P.; Beratan D. N.; Yang W. NCIPLOT: A Program for Plotting Noncovalent Interaction Regions. J. Chem. Theory Comput. 2011, 7, 625–632. 10.1021/ct100641a. 21516178 PMC3080048

