# Supplementary material for: Importance of Van der Waals Interactions in Hydrogen Adsorption on a Silicon-carbide Nanotube Revisited with vdW-DFT and Quantum Monte Carlo
Source: ACS Omega. 2021 Sep 16;6(38):24630–6. doi: 10.1021/acsomega.1c03318 (PMC8482461; doi:10.1021/acsomega.1c03318)
Supplement: Supplementary file 1 — ao1c03318_si_001.pdf [file ao1c03318_si_001.pdf]

Importance of van der Waals interactions in hydrogen adsorption on a silicon-carbide nanotube revisited with vdW-DFT and quantum Monte Carlo: Supporting Information

Genki I. Prayogo,*,† Hyeondeok Shin,‡ Anouar Benali,‡ Ryo Maezono,† and Kenta Hongo*,¶

† *School of Information Science, JAIST, Asahidai 1-1, Nomi, Ishikawa, 923-1292, Japan*

‡ *Computational Science Division, Argonne National Laboratory, Argonne, Illinois 60439, USA*

¶ *Research Center for Advanced Computing Infrastructure, JAIST, Asahidai 1-1, Nomi, Ishikawa 923-1292, Japan*

E-mail: g.prayogo@icloud.com; kenta_hongo@mac.com

In interpreting the DMC results, it is important to discuss about the extrapolations used in the removal of the finite size error and timestep biases, especially in a noncovalent system, such as the system studied, where the magnitude of the interaction energies are relatively small. Indeed, for the SiCNT+H_2 complex, the absolute DMC energy between the smallest 2 unit and the largest 8 unit supercell can differ by up to 15.82 kcal/mol, which translates to ~0.72 kcal/mol for each atom within the unit cell, or around 52% of the target interaction energy. Despite this seemingly large size, we found the error to be reasonably well-behaved, and such was easily controlled by performing adequate statistical accumulation and the linear

extrapolation by $1/N$ of the system size, resulting in final uncertainties of not more than $\sim$0.14 kcal/mol for each H_2 distance. Due to the presence of open boundaries at the x and y plane (perpendicular to the SiCNT axis), we were not able to exploit the often used Chiesa correction[1] to reduce the finite size error. The timestep bias on the other hand is relatively small, with the maximum of $\sim$0.25 kcal/mol an atom for the largest 0.04 a.u.$^{-1}$ timestep size. There is a trade-off between choosing a smaller DMC timestep and largest simulation cell and the computational cost, this being the limitation of the current calculation.

1 Timestep Extrapolations

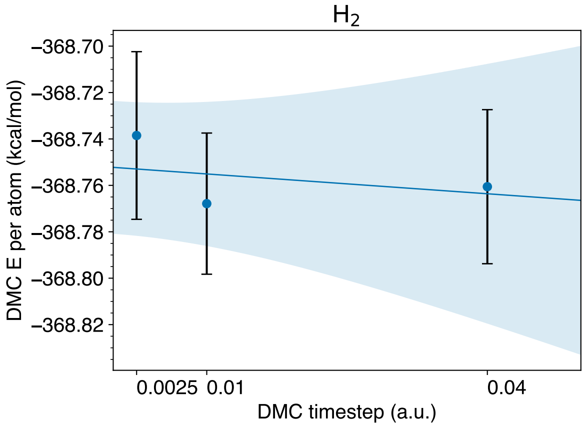

Figure 1

Figure 2: DMC timestep extrapolations for H_2.

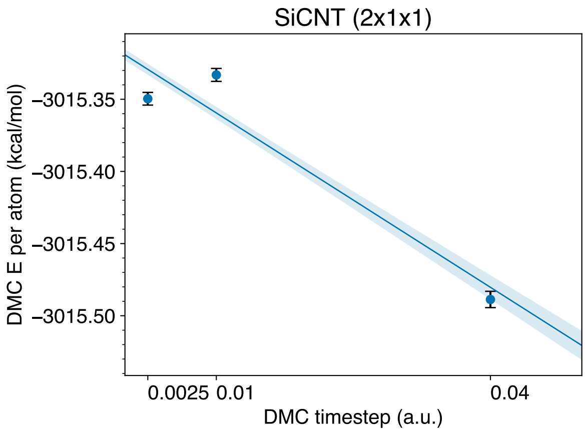

(a)

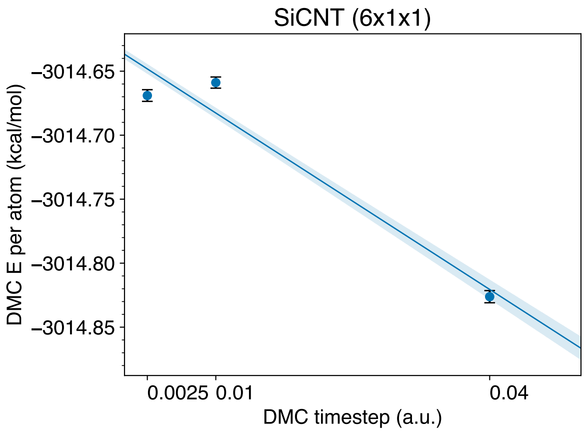

(b)

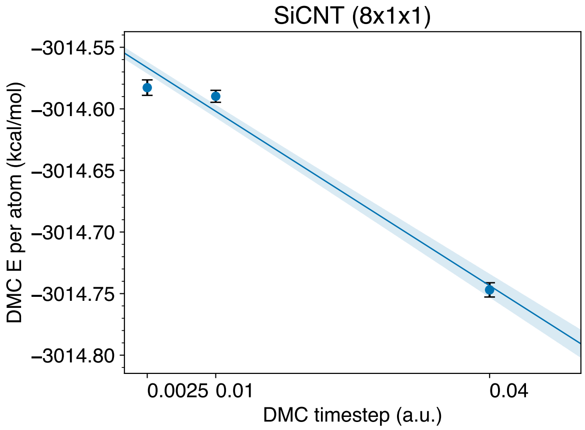

(c)

Figure 3: DMC timestep extrapolations for SiCNT-only fragment with (a) 2×1×1, (b) 6×1×1, and (c) 8×1×1 supercell.

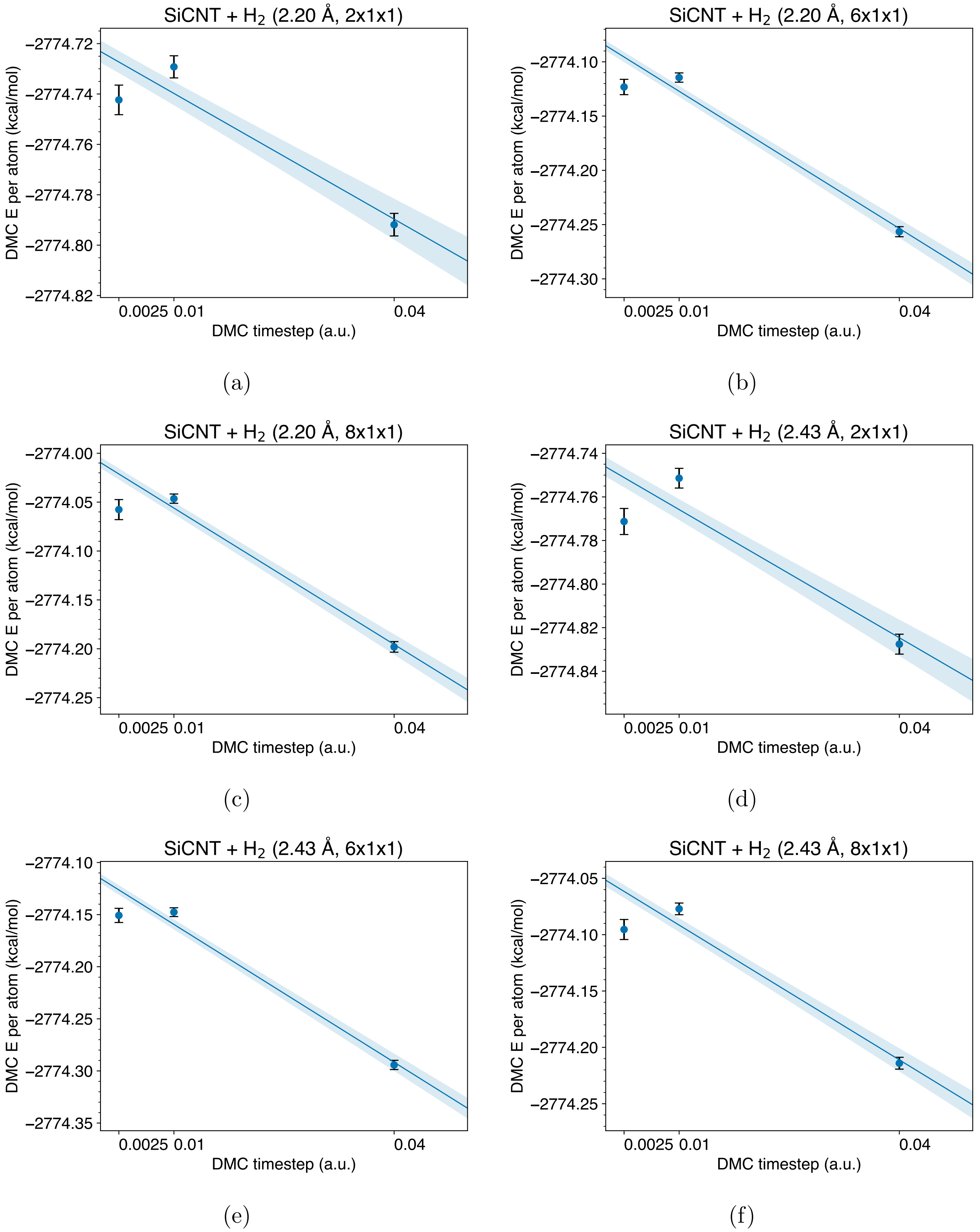

Figure 4: DMC timestep extrapolations for SiCNT+H_2 complex, at 2.20 Å distance with (a) 2×1×1, (b) 6×1×1, and (c) 8×1×1 supercell, and at 2.43 Å with (d) 2×1×1, (e) 6×1×1, and (f) 8×1×1 supercell.

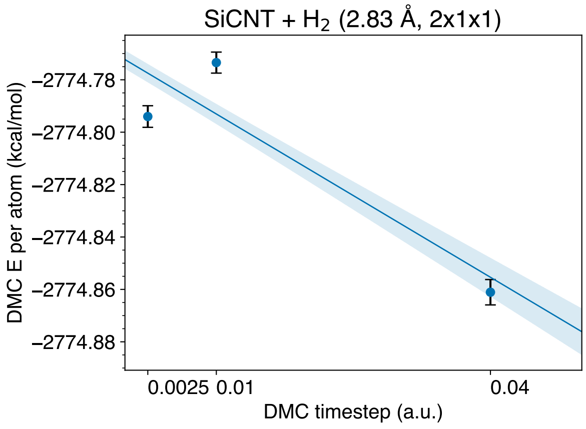

(a)

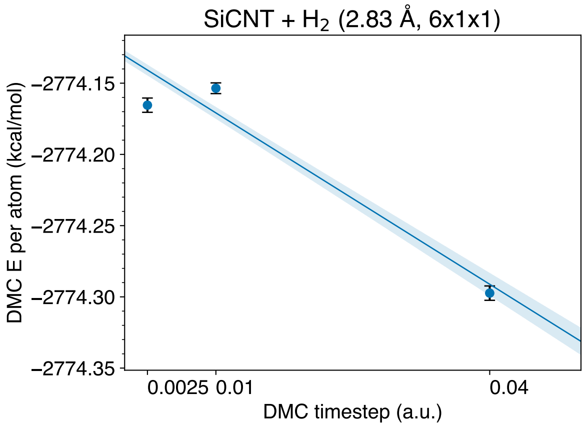

(b)

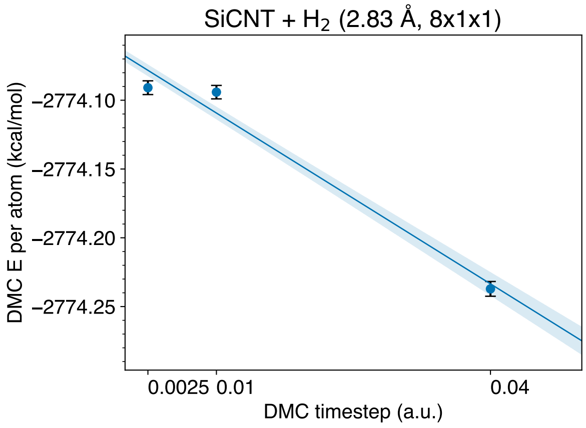

(c)

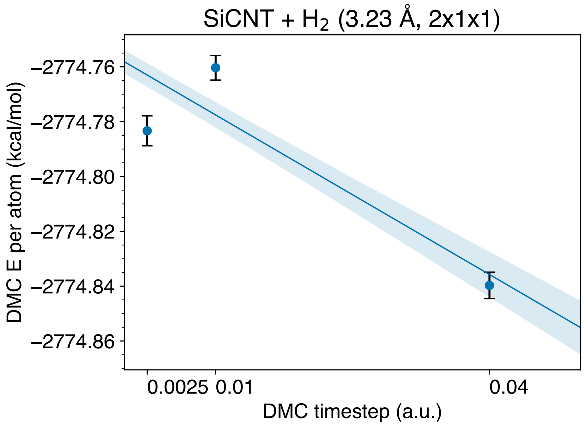

(d)

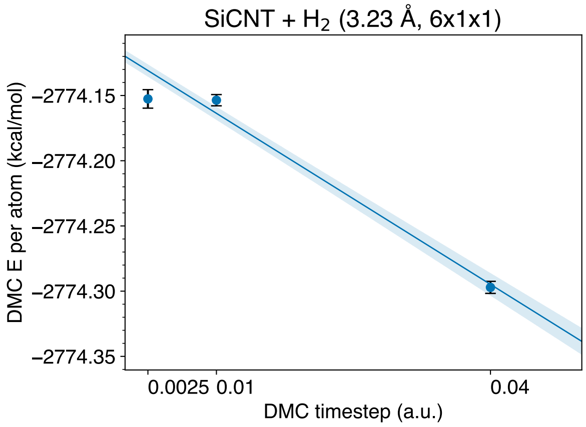

(e)

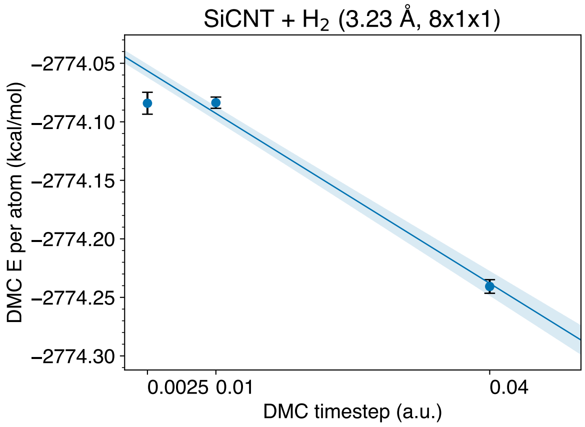

(f)

Figure 5: DMC timestep extrapolations for SiCNT+H_2 complex, at 2.83 Å distance with (a) 2×1×1, (b) 6×1×1, and (c) 8×1×1 supercell, and at 3.23 Å with (d) 2×1×1, (e) 6×1×1, and (f) 8×1×1 supercell.

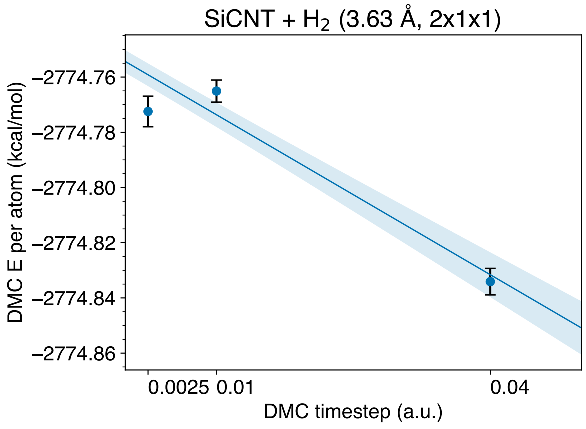

(a)

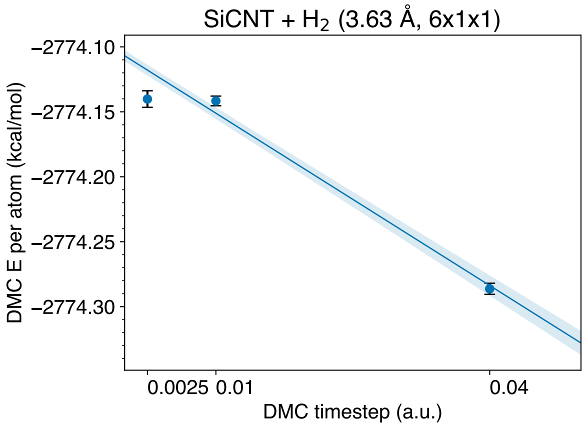

(b)

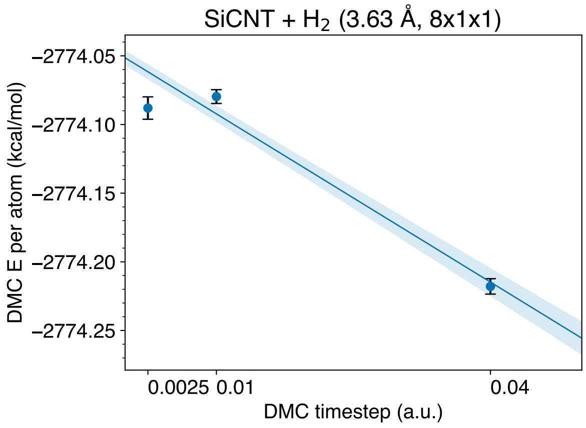

(c)

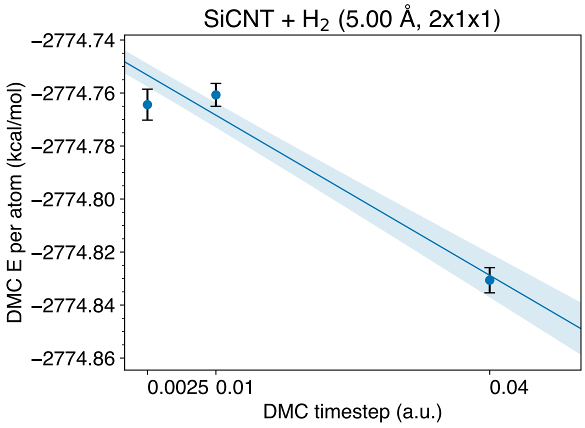

(d)

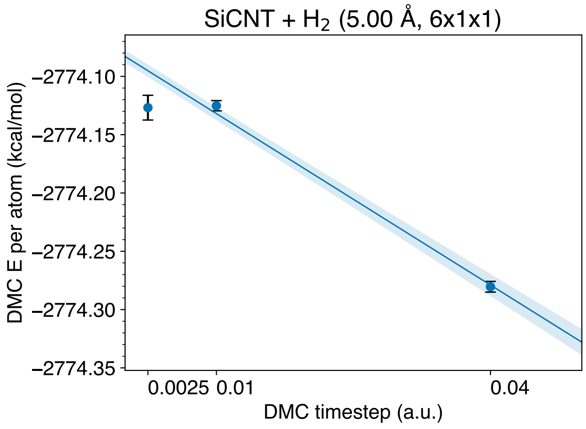

(e)

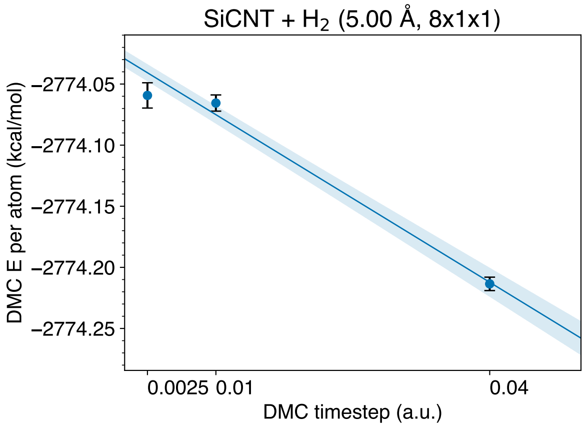

(f)

Figure 6: DMC timestep extrapolations for SiCNT+H_2 complex, at 3.63 Å distance with (a) 2×1×1, (b) 6×1×1, and (c) 8×1×1 supercell, and at 5.00 Å with (d) 2×1×1, (e) 6×1×1, and (f) 8×1×1 supercell.

2 Finite Size Corrections

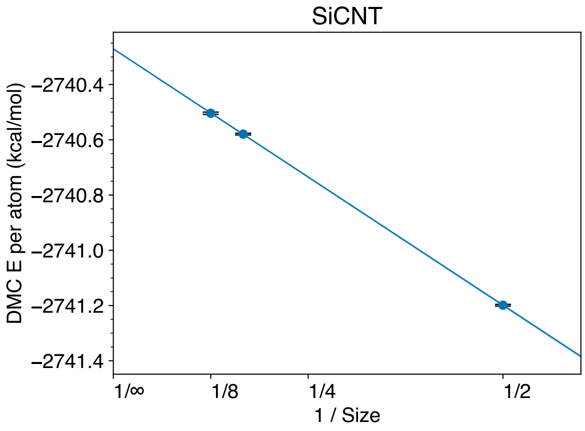

Figure 7: DMC finite size correction for SiCNT-only fragment.

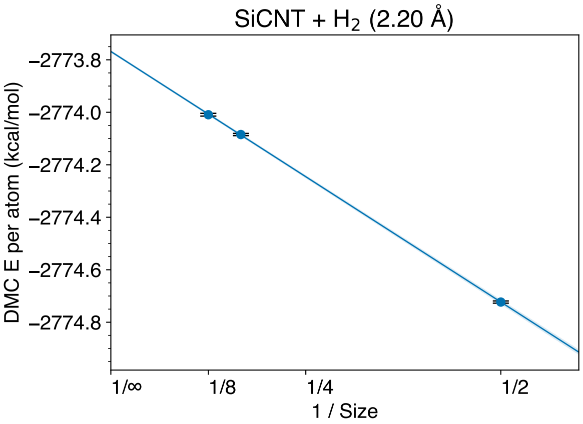

(a)

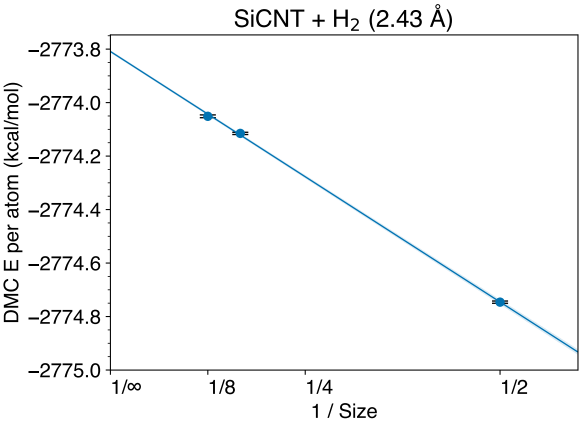

(b)

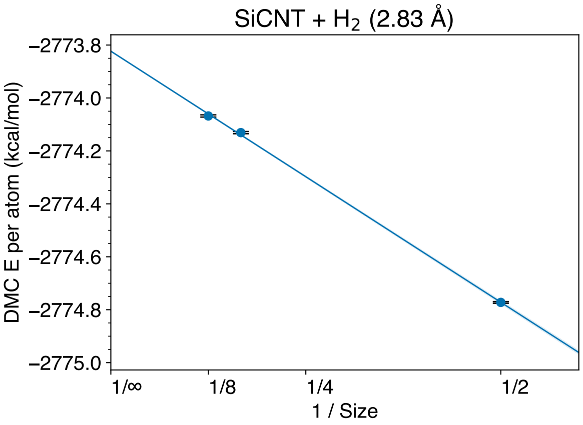

(c)

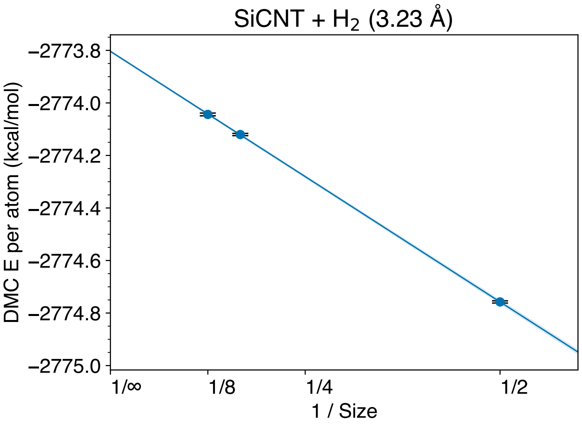

(d)

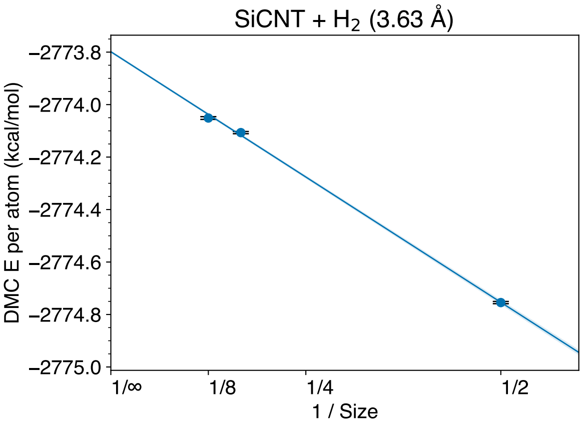

(e)

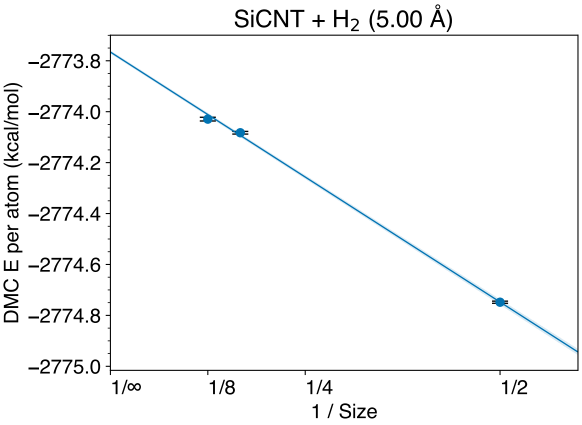

(f)

Figure 8: DMC finite size correction for SiCNT+H_2 complex, at (a) 2.20 Å, (b) 2.43 Å, (c) 2.83 Å, (d) 3.23 Å, (e) 3.63 Å, and (f) 5.00 Å distance.

References

(1) Chiesa, S.; Ceperley, D. M.; Martin, R. M.; Holzmann, M. Finite-Size Error in Many-Body Simulations with Long-Range Interactions. *Phys. Rev. Lett.* **2006**, *97*, 076404.
